# Bandwidth and gain enhancement of composite right left handed metamaterial transmission line planar antenna employing a non foster impedance matching circuit board

**DOI:** 10.1038/s41598-021-86973-x

**Published:** 2021-04-02

**Authors:** Mohammad Alibakhshikenari, Bal S. Virdee, Ayman A. Althuwayb, Leyre Azpilicueta, Naser Ojaroudi Parchin, Chan H. See, Raed A. Abd-Alhameed, Francisco Falcone, Isabelle Huynen, Tayeb A. Denidni, Ernesto Limiti

**Affiliations:** 1 Electronic Engineering Department, University of Rome “Tor Vergata”, Via Del Politecnico 1, 00133 Rome, Italy; 2 Center for Communications Technology, London Metropolitan University, London, N7 8DB UK; 3 Department of Electrical Engineering, Jouf University, Sakaka, 72388 Aljouf Saudi Arabia; 4 School of Engineering and Sciences, Tecnologico de Monterrey, 64849 Monterrey, Mexico; 5 Faculty of Engineering and Informatics, University of Bradford, Bradford, BD7 1DP West Yorkshire UK; 6 School of Engineering and the Built Environment, Edinburgh Napier University, 10 Colinton Rd, Edinburgh, EH10 5DT UK; 7 Electronic and Communication Engineering Department, Public University of Navarre, 31006 Pamplona, Spain; 8 Institute of Smart Cities, Public University of Navarre, 31006 Pamplona, Spain; 9 Institute of Information and Communication Technologies, Electronics and Applied Mathematics, Université Catholique de Louvain, 1348 Louvain-la-Neuve, Belgium; 10 Institut National de La Recherche Scientifique (INRS), University of Quebec, Montréal, Québec H5A 1K6 Canada

**Keywords:** Engineering, Electrical and electronic engineering

## Abstract

The paper demonstrates an effective technique to significantly enhance the bandwidth and radiation gain of an otherwise narrowband composite right/left-handed transmission-line (CRLH-TL) antenna using a non-Foster impedance matching circuit (NF-IMC) without affecting the antenna’s stability. This is achieved by using the negative reactance of the NF-IMC to counteract the input capacitance of the antenna. Series capacitance of the CRLH-TL unit-cell is created by etching a dielectric spiral slot inside a rectangular microstrip patch that is grounded through a spiraled microstrip inductance. The overall size of the antenna, including the NF-IMC at its lowest operating frequency is 0.335λ_0_ × 0.137λ_0_ × 0.003λ_0_, where λ_0_ is the free-space wavelength at 1.4 GHz. The performance of the antenna was verified through actual measurements. The stable bandwidth of the antenna for |S_11_|≤ − 18 dB is greater than 1 GHz (1.4–2.45 GHz), which is significantly wider than the CRLH-TL antenna without the proposed impedance matching circuit. In addition, with the proposed technique the measured radiation gain and efficiency of the antenna are increased on average by 3.2 dBi and 31.5% over the operating frequency band.

## Introduction

Highly reliable and portable ultra-high frequency (UHF) wireless communication systems mainly employ monopole or dipole antennas. As the physical size of the antenna is proportional its operating wavelength, UHF-band antennas can have a length between 0.1 to 1 m. Various types of small UHF-band antennas have been previously proposed; however, they cannot be adapted for conformal installations and/or their bandwidth is limited^1–3^. Another method to reduce the antenna size is using metamaterials (MTMs) or left-handed (LH) ($\varepsilon <0$, $\mu <0$) materials^4–6^. In practice, when creating a MTM the parasitic elements associated with transmission-lines (TLs), i.e. right-handed (RH) ($\varepsilon >0$, $\mu >0$), combine with the LH characteristics to create a MTM structure commonly referred to as composite right/left-handed transmission-line (CRLH-TL)^7^. Antennas based on CRLH-TLs can be designed to operate in different modes, such as the zeroth-order resonance (ZOR) mode, + 1, and − 1 modes^8,9^. The zeroth-order resonance frequency makes CRLH-TLs independent of its physical dimensions. This property is taken advantage of here to miniaturize the antenna. As CRLH-TL can be easily realized using standard printed circuit board (PCB) technologies they have been used in the design of multiband antennas^10^, frequency reconfigurable antennas^11^ and pattern reconfigurable antennas^8^. However, the inherent narrow bandwidth of CRLH-TLs limits the range of applications of this type of antenna.

To overcome the bandwidth limitation of CRLH-TL antenna, it is necessary to introduce an active device that presents a negative reactance that counteracts the positive reactance of the antenna. This is achieved here with the use of a non-Foster impedance matching circuit. The challenging aspect of NF-IMC design is a stability-performance tradeoff, where instability leads to oscillation or latching. Optimal performance is achieved at the stability threshold, leaving little margin for error due to imperfect modeling, fabrication tolerances, and environmental changes. Numerous designs of the non-Foster circuit have been previously reported to realize wideband matching of electrically small antennas^12–15^. The improvement in the matching performance reported using NF-IMC is mainly in terms of reflection-coefficient, which does not completely characterize the antenna’s operational band.

In this paper we demonstrate that by combining the proposed non-Foster impedance matching circuit (NF-IMC) with a narrowband CRLH-TL microstrip antenna it is possible to significantly enhance the antenna’s bandwidth and radiation gain performance without compromising its stability. This is due to the negative reactance created by the NF-IMC that is used to cancel the antenna’s high and positive reactance over its operating frequency range. The proposed antenna described here is implemented with six unit-cells of CRLH-TL, where the equivalent circuit of each unit-cell consists of series left-handed capacitance and shunt spiral left-handed inductance. The size of the proposed antenna is 56 $\times$ 20 $\times$ 0.8 mm^3^ or 0.261 ${\uplambda }_{0}\times$ 0.093 ${\uplambda }_{0}\times$ 0.003 ${\uplambda }_{0}$, where ${\uplambda }_{0}$ is at the minimum operation frequency of the antenna which is 1.4 GHz. The size of the non-Foster circuit is 0.074 ${\uplambda }_{0}\times$ 0.044 ${\uplambda }_{0}$ (16 $\times$ 9.5 mm^2^). The proposed antenna has a measured bandwidth of > 1 GHz across 1.4–2.45 GHz for |S_11_|≤ − 18 dB, which is significantly wider than the CRLH-TL antenna without the non-Foster circuitry. With NF-IMC the measured antenna's radiation gain is shown to improve over its operating frequency range on average by approximately 3 dB.

## Design of non-foster impedance matching circuit

**Figure 1:**
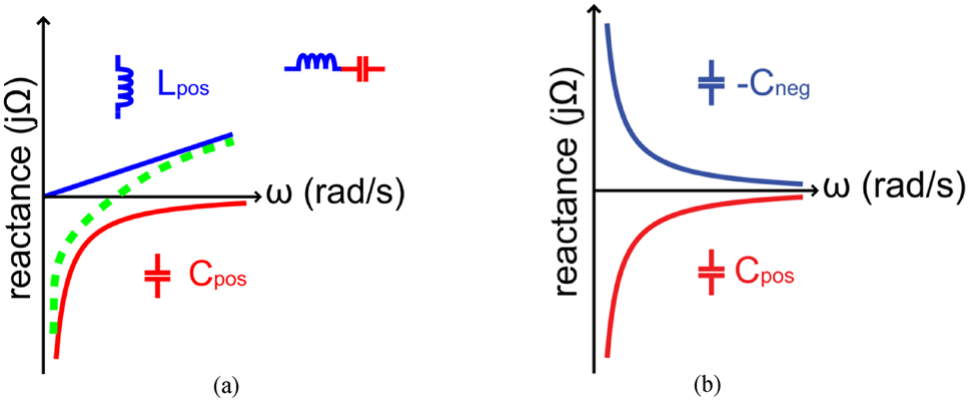
Matching with (**a**) Foster impedance, and (**b**) non-Foster impedance.

**Figure 2:**
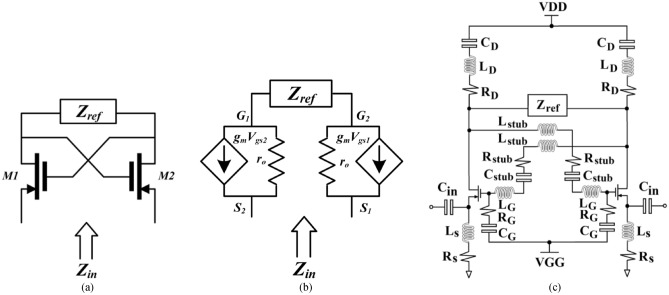
Non-Foster impedance matching circuit (NF-IMC) obtained through optimization using ADS version 2019 from Keysight Technologies. (**a**) Basic topology, (**b**) equivalent small-signal model, and (**c**) equivalent circuit of the optimized NF-IMC design used here.

Impedance matching circuits (IMCs) using conventional passive elements such as a *LC-*circuit are applicable only for narrowband devices. To circumvent this issue the negative impedance property of NF-IMC is exploited here to reverse the reactance slope of conventional *LC*-circuits, as illustrated in Fig. 1, to thereby accomplish impedance matching over a wider band. The design of the NF-IMC is based on the generic principle described in^16^ involving the use of cross-coupled transistors, as shown in Fig. 2a. This circuit is utilized here to convert the load impedance (*Z*_*ref*_) seen by the two transistors to its negative equivalent. Positive feedback loop is created by cross coupling the gate and drain of the two transistors, which is typically used to design an oscillator circuit. Therefore, avoiding oscillations and ensuring stability are critical in the design of the NF-IMC. In the cross-coupled transistor arrangement the phase of the current at the input and output are identical, but there is a phase difference between the input and output voltages of 180°. From the small-signal model of the simplified NF-IMC topology in Fig. 2b, it can be shown that

1 $${Z}_{in}=\frac{2{r}_{o}}{1+{g}_{m}{r}_{o}}+\frac{{Z}_{ref}}{1+{g}_{m}{r}_{o}}-\frac{{Z}_{ref}{g}_{m}{r}_{o}}{1+{g}_{m}{r}_{o}}$$

where ${r}_{o}$ and ${g}_{m}$ are the transistor’s output resistance and transconductance, respectively. Assuming ${g}_{m}{r}_{o}\gg 1$ and is large compared to ${Z}_{ref}$, (1) can be approximated as ${Z}_{in}={-Z}_{ref}.$ The actual NF-IMC used in Fig. 2c was obtained through optimization using ADS from Keysight Technologies to provide impedance matching from 1.4 to 2.45 GHz. The optimized parameters of the NF-IMC are given in Table 1. Avago ATF-53189 transistor with an operating frequency of 50–6500 MHz was used here. The bias voltage V_DD_ is 2 V.

**Table 1:** Parameters of the non-Foster impedance matching circuit (NF-IMC).

Parameter	Value
${Z}_{ref}$	13 pF
${C}_{D}$	125 pF
${L}_{D}$	150 nH
${R}_{D}$	700 Ω
${C}_{stub}$	8.5 pF
${R}_{stub}$	50 Ω
${L}_{stub}$	12 nH
${C}_{in}$	50 pF
${L}_{S}$	150 nH
${R}_{S}$	700 Ω
${C}_{G}$	125 pF
${L}_{G}$	150 nH
${R}_{G}$	700 Ω

**Figure 3:**
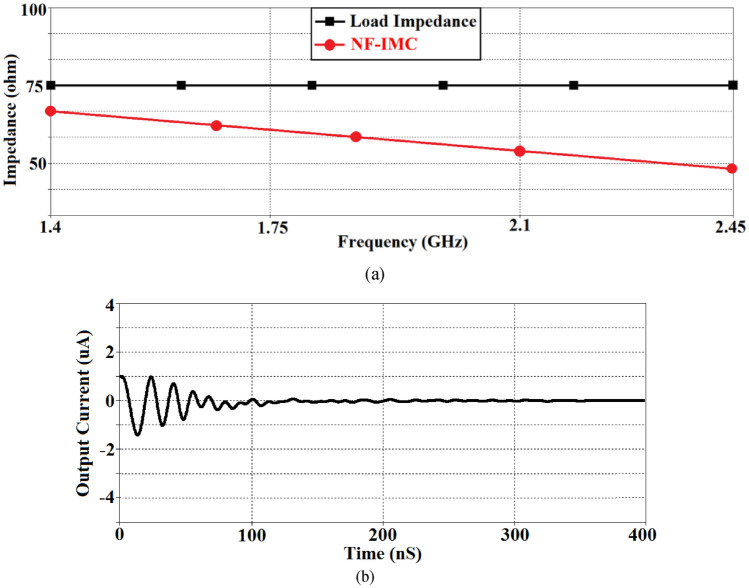
(**a**) Impedance of the proposed non-Foster matching circuit and load impedance (*Z*_*ref*_), and (**b**) time domain transient response of the NF-IMC.

Active feedback is necessary in NF-IMC to realize the desired negative impedance. However, NF-IMCs can generate positive feedback when connected to the antenna system which can adversely affect its stability. As NF-IMC’s delicate stability is vulnerable to environmental changes, a small perturbation in antenna’s impedance could increase the positive feedback and make the NF-IMCs deviate from the stable condition into a large signal oscillator. It was therefore important to establish the stability of the NF-IMC. Stability over a wide band was achieved by cross coupling the transistors with series *LC* circuit. The stability was confirmed by checking impedance of the NF-IMC and the time domain transient response, as shown in Fig. 3. The stability was maintained by making the impedance of the non-Foster matching circuit smaller than the impedance of the antenna, which is confirmed in Fig. 3a over 1.4–2.45 GHz. Figure 3b shows the time domain transient response of the NF-IMC is stable after 0.2 microseconds of switching ‘on’ the NF-IMC.

## Design of proposed CRLH-TL antenna

The equivalent circuit of a typical CRLH-TL unit-cell is constituted from a left-handed (LH) and right-handed (RH) circuitry^7^, where the LH circuit is composed of the series capacitors (${C}_{L}$) and the shunt inductors (${L}_{L}$). In practice, the unavoidable parasitic reactance elements are introduced in transmission-line structure, in particular series RH inductances (${L}_{R}$) and the parasitic shunt RH capacitances (${C}_{R}$) which compromise the existence of the purely LH circuit. The zeroth-order resonant frequency of the CRLH-TL is affected by these parasitic effects. Below the zeroth-order resonant frequency, CRLH-TL structure exhibits LH characteristics, and above zeroth-order resonant frequency, CRLH-TL exhibits RH characteristics. At the zeroth-order frequency, the resonant frequency is independent of the physical size. This property is exploited here to miniaturize the antenna. The zeroth-order resonant frequency is controlled by the series and shunt resonant frequencies defined by

2 $${\omega }_{se}=1/\sqrt{{L}_{R}{C}_{L}}$$

3 $${\omega }_{sh}=1/\sqrt{{L}_{L}{C}_{R}}$$

The propagation constant (*β*) for a CRLH unit-cell is given by

4 $$\beta =S\left(\omega \right)\sqrt{{\omega }^{2}{L}_{R}{C}_{R}+\frac{1}{{\omega }^{2}{L}_{L}{C}_{L}}-\left(\frac{{L}_{R}}{{L}_{L}}+\frac{{C}_{R}}{{C}_{L}}\right)}$$

where

5 $$S\left(\omega \right)=\left\{\begin{array}{c}-1~\text{if}~ \omega <{\omega }_{\text{sh}}=min\left(\frac{1}{\sqrt{{L}_{R}{C}_{L}}}, \frac{1}{\sqrt{{L}_{L}{C}_{R}}}\right)\\ +1~\text{if}~ \omega <{\omega }_{\text{se}}=max\left(\frac{1}{\sqrt{{L}_{R}{C}_{L}}}, \frac{1}{\sqrt{{L}_{L}{C}_{R}}}\right)\end{array}\right.$$

**Figure 4:**
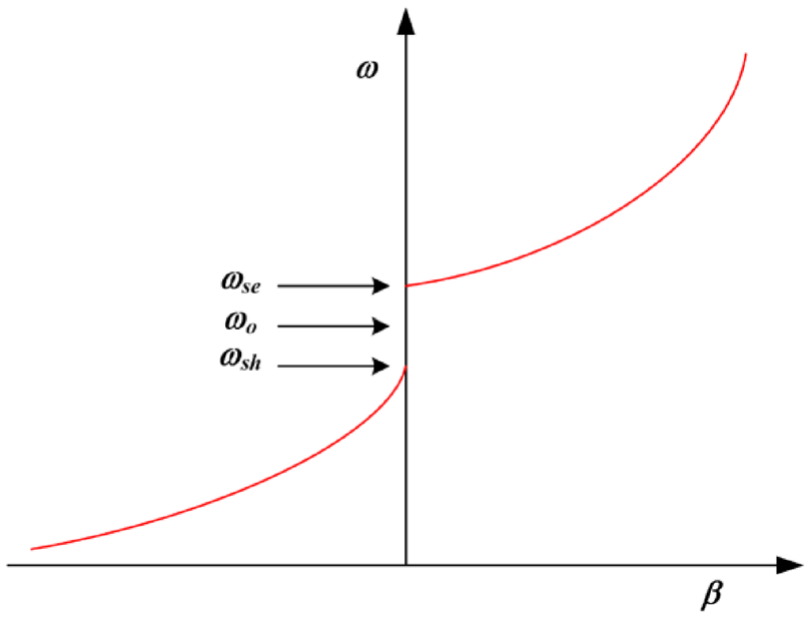
Dispersion curve of CRLH-TL unit-cell.

Figure 4 shows the dispersion diagram of a typical CRLH-TL unit-cell when ω_*se*_ and ω_*sh*_ are unequal. At these resonant frequencies (ω_*se*_ and ω_*sh*_) where *β* = 0, an infinite wavelength can be supported. According to the transmission-line theory CRLH-TL resonates at a frequency given by ^7^

6 $${\beta }_{n}=\frac{n\pi }{l} \left(n=0, \pm 1,\pm 2,\dots ., \pm \left(N-1\right)\right)$$

where, *l, n* and *N* are the physical length of the resonator, mode number, and number of unit cells, respectively. When *n* is zero, the wavelength, *λ*_*g*_ = 2π/|*β*_*n*_|, becomes infinite and the resonant frequency of the zeroth-order mode becomes independent of the size of the resonator.

**Figure 5:**
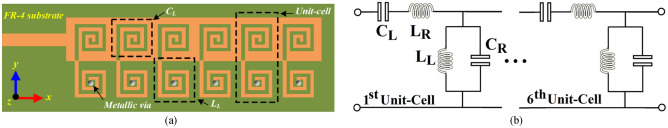
The proposed CRLH-TL antenna, (**a**) layout, realized in CST Microwave Studio version 2019, and (**b**) equivalent circuit, obtained through optimization using ADS version 2019.

**Figure 6:**
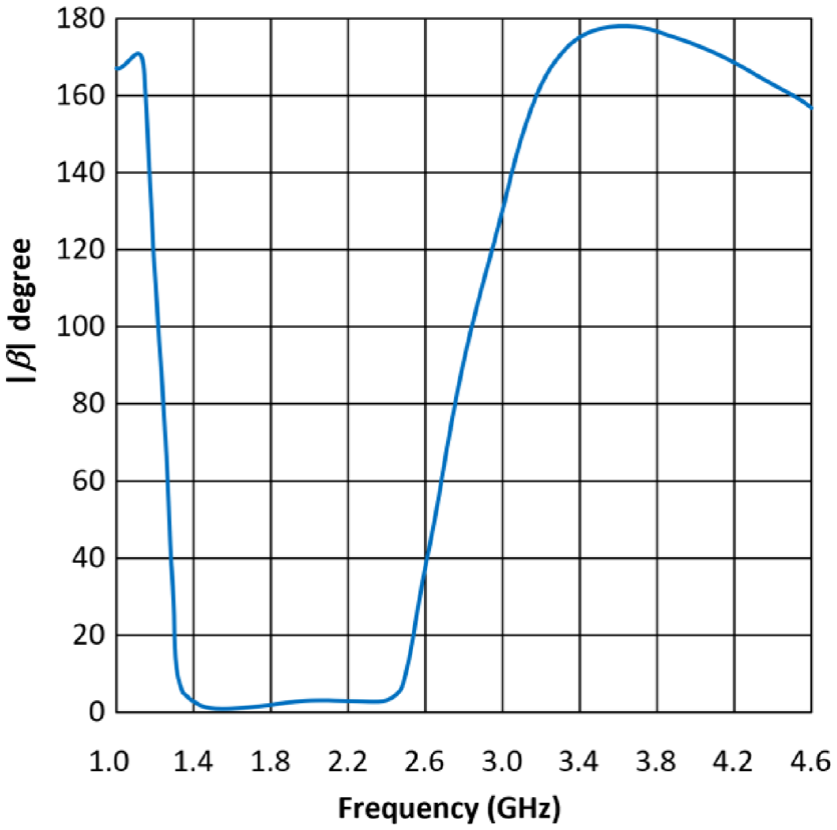
Dispersion diagram of the proposed CRLH-TL unit-cell.

**Figure 7:**
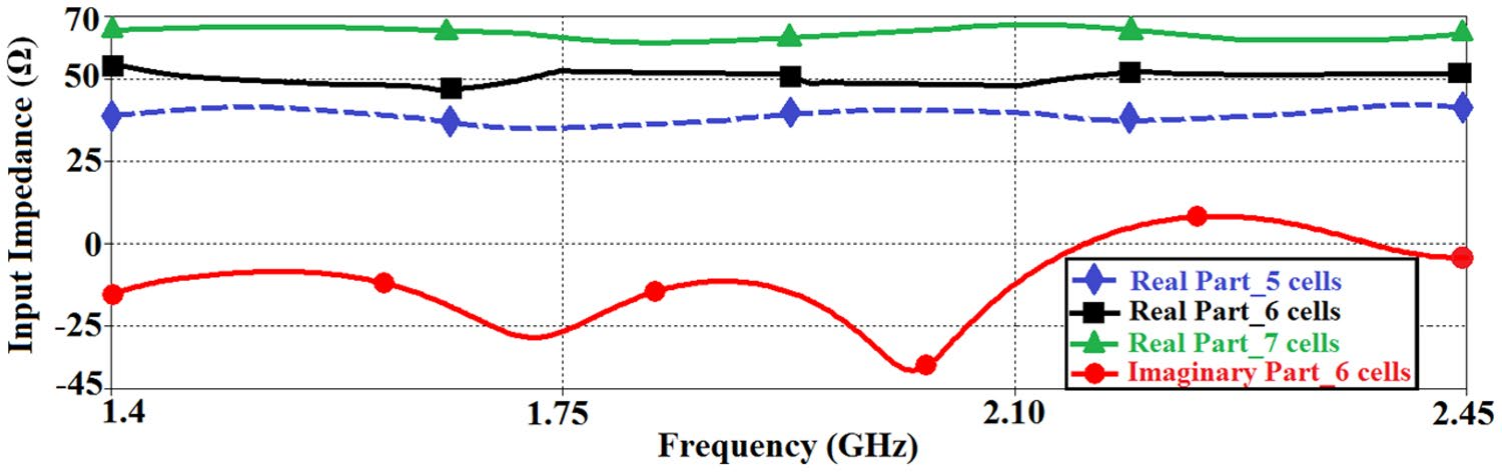
Input impedance of the CRLH-TL antenna as a function of number of unit-cells.

The antenna was designed by determining the reactance values in Eqs. (2) & (3) such that each of the two zeroth-order resonant frequencies define the boundary of the frequency range of interest. This was done by using the optimization tool in ADS by Keysight Technologies. The CRLH-TL unit-cell parameters were then converted to their physical implementation on microstrip technology, where ${C}_{L}$ is implemented by etching a spiral slot on a rectangular patch, and the ${L}_{L}$ is realized using a high impedance spiraled microstrip-line that is grounded with a metalized via-hole. The inductor was spiraled purely to minimize the spaced used. As mentioned earlier the parasitic RH elements (${C}_{R}$ & ${L}_{R}$) are unavoidable components in the transmission-line structure due to existence of the voltage gradient between the patch and ground plane, and the currents flowing on the surfaces, respectively^13^. As these parameters effect the overall frequency range of the antenna it was therefore necessary to optimize the structural parameters of the antenna unit-cell using ADS to realize the desired performance. The physical layout of the proposed antenna, which consists of a cascade of six unit-cells, and its equivalent circuit are shown in Fig. 5. The dispersion diagram of the CRLH-TL unit-cell depicted in Fig. 6 shows the LH and RH regions are 1.14 GHz < *f*_*LH*_ < 1.41 GHz and 2.45 GHz < *f*_*RH*_ < 3.6 GHz, respectively. It also shows the propagation constant of the CRLH-TL unit-cell is near zero in the frequency range of interest between 1.4 and 2.46 GHz. The simulation analysis in Fig. 7 reveals show the input impedance of the antenna is affected by the number of unit-cells. The antenna was implement using six unit-cells so that its impedance was compatible with the 50Ω NF-IMC board.

The proposed CRLH-TL antenna was constructed on the FR-4 lossy substrate with ${\varepsilon }_{r}$=4.4, thickness *h* = 0.8 mm, and tan $\delta$=0.025. The equivalent circuit parameters of the CRLH-TL unit-cell were extracted using the pseudo-inverse method^17^ from the measured *S*-parameters. This involved converting the *S*-parameters to *ABCD*-parameters and the corresponding impedance and admittance from which the values of the lumped elements were obtained. The averaged values of the unit-cell parameters over the frequency of interest (1.4–2.45 GHz) are: ${L}_{L}=0.39 nH$, ${C}_{L}=6.12 pF$, ${L}_{R}=2.11 nH$, and ${C}_{R}=10.74 pF$. The structural parameters of the optimized antenna are given in Table 2, where ${\uplambda }_{0}$ is the free-space wavelength at 1.4 GHz.

**Table 2:** Structural Parameters of the CRLH-TL Antenna.

Number of spiral slot turns	3
Width of spiral slots	0.75 mm
Gap between spiral slots	0.75 mm
Gap between the unit-cells	2.25 mm
Number of inductive spiral turns	3
Width of inductive spiral	0.75 mm
Gap between the inductive spirals	0.75 mm
Radius of the via-hole	0.4 mm
Number of unit-cells	6
Unit-cell dimensions	7 × 16 × 0.8 mm^3^ (0.032 ${\uplambda }_{0}\times$ 0.074 ${\uplambda }_{0}\times$ 0.003 ${\uplambda }_{0})$
Antenna dimensions	56 × 20 × 0.8 mm^3^ (0.261 ${\uplambda }_{0}\times$ 0.093 ${\uplambda }_{0}\times$ 0.003 ${\uplambda }_{0})$
Ground-plane dimensions	56 × 20 × 0.035 mm^3^
Dimensions of feedline	13 × 2.3 mm^2^
Dimensions of NF-IMC board	16 × 9.5 mm^2^

**Figure 8:**
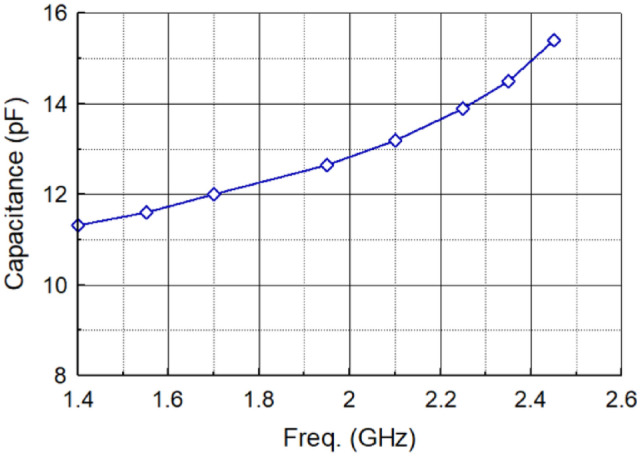
Input capacitance of the antenna as a function of frequency.

The input capacitance of the antenna is approximated by $Imag \left({Z}_{in}\right)=1/j\omega C$. The antenna’s input capacitance as a function of frequency is shown in Fig. 8. The input capacitance of the antenna was counteracted with the negative reactance generated by the NF-IMC and thereby overcome the inherent narrowband characteristic of the antenna.

## Fabricated prototype and measurements

**Figure 9:**
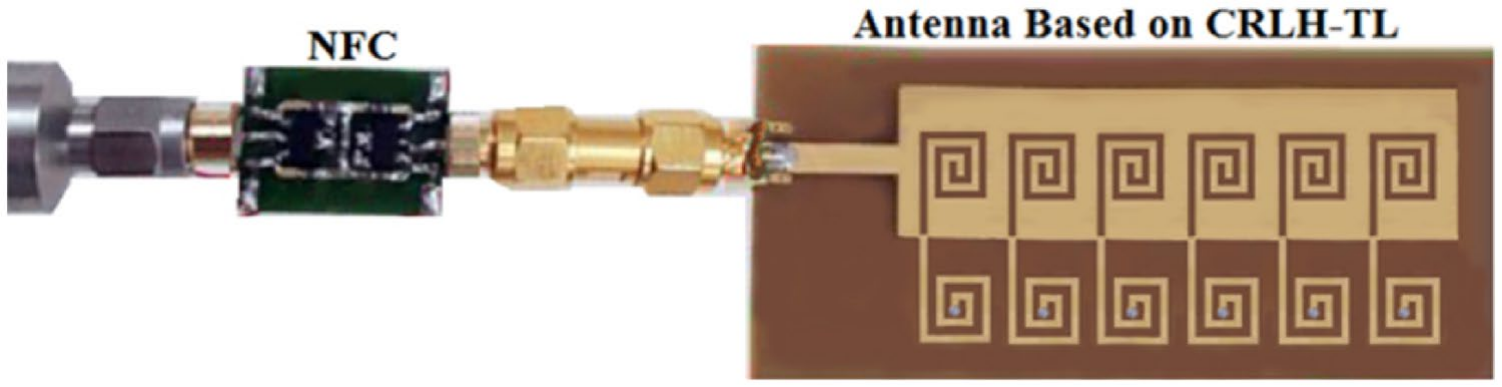
Fabricated CRLH-TL antenna prototype connected to NF-IMC board.

**Figure 10:**
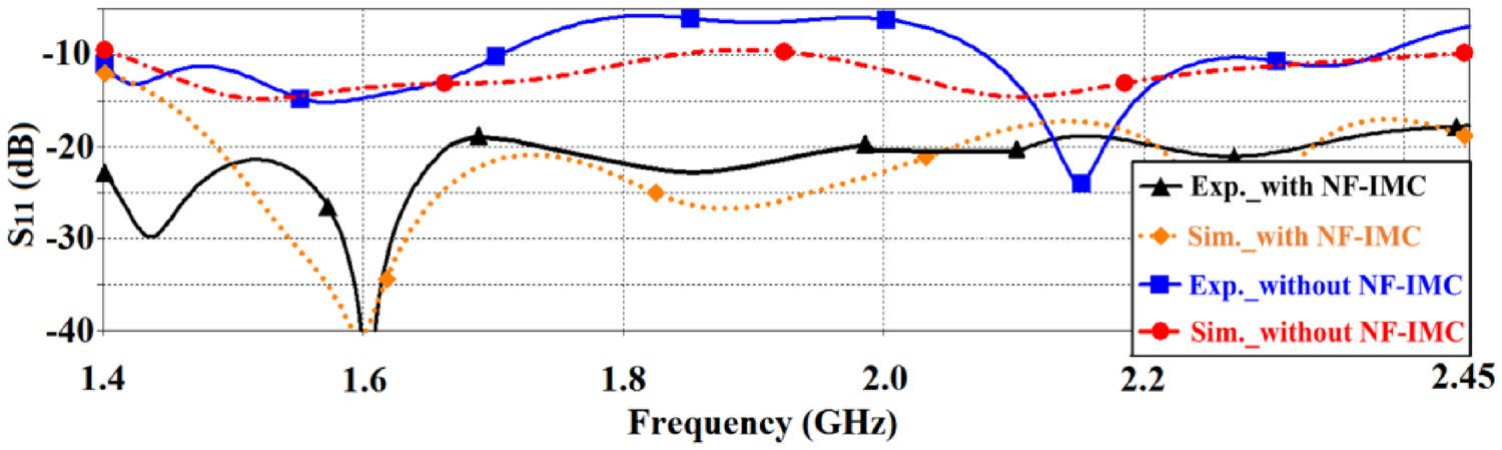
Simulated and measured reflection-coefficient response of the proposed CRLH-TL antenna without and with NF-IMC.

The fabricated prototype of the proposed antenna based on CRLH-TL is combined with the NF-IMC as shown in Fig. 9. An SMA male to SMA male coupler is used to connect the antenna to NF-IMC board. With the antenna connected to the NF-IMC board the measured reflection-coefficient is better than − 18 dB across 1.4–2.45 GHz, which corresponds to a fractional bandwidth of 54.54%. Simulated and measured reflection-coefficient responses of the proposed antenna before and after applying NF-IMC are shown in Fig. 10. Although there is reasonably good correlation between the simulated and measured results the large disparity between 1.4 to 1.5 GHz is attributed to the manufacturing tolerances and unaccounted mismatch with the SMA male to SMA male coupler.

**Figure 11:**
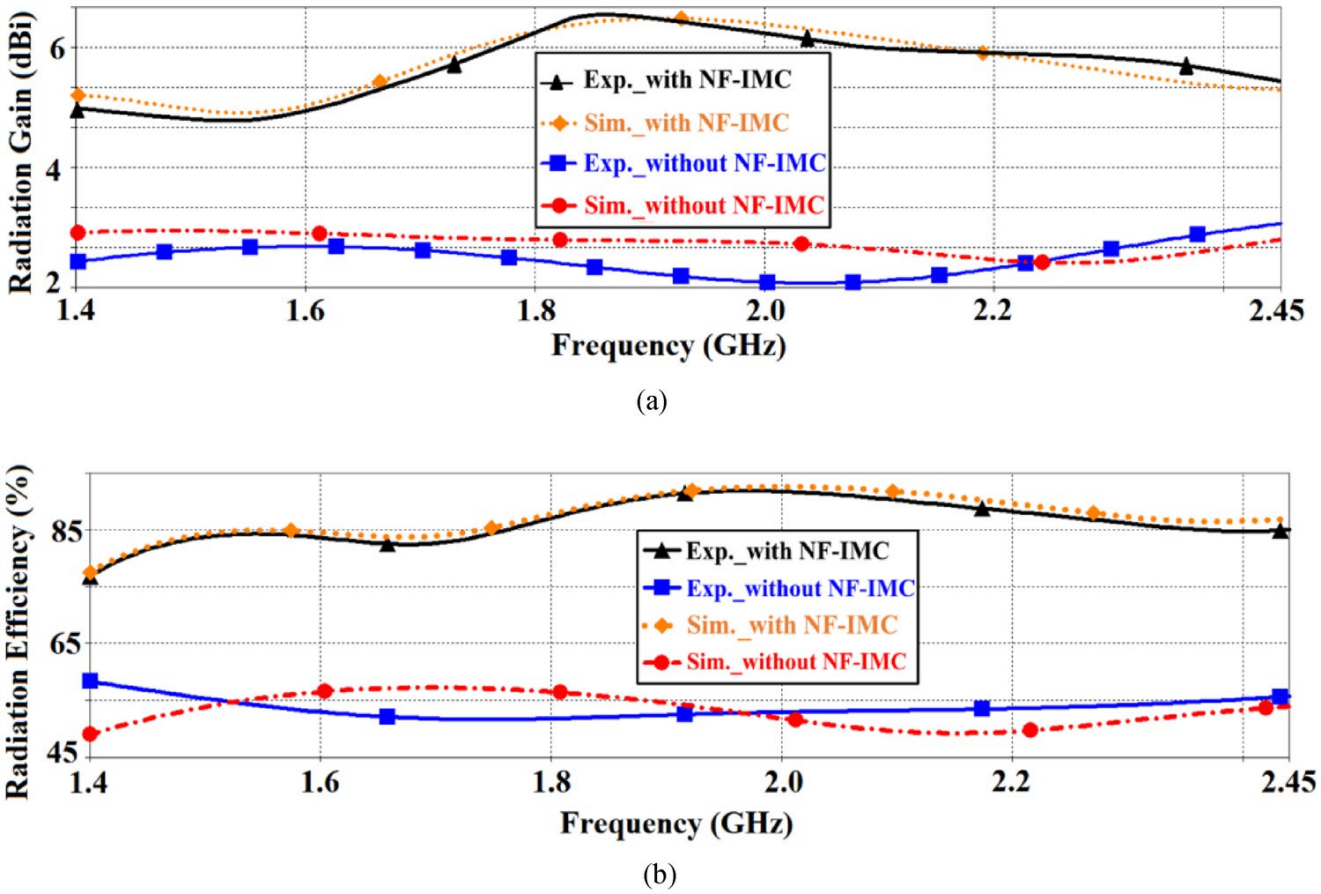
Simulated and measured radiation properties of the proposed CRLH-TL antenna with and without NF-IMC in the *xy*-plane, (**a**) gain, and (**b**) efficiency.

The simulated and measured radiation gain and efficiency in the *xy*-plane (E-plane) of the proposed antenna before and after applying NF-IMC are shown in Fig. 11. There is good correlation between the simulated and measured results. It is evident from these results that with NF-IMC the measured radiation gain increases by 3.2 dBi from an average of 2.4 dBi to 5.6 dBi across 1.4–2.45 GHz. Over the same frequency span the measured radiation efficiency of the antenna increased by 31.5% from an average of 54.5% to 86%.

**Figure 12:**
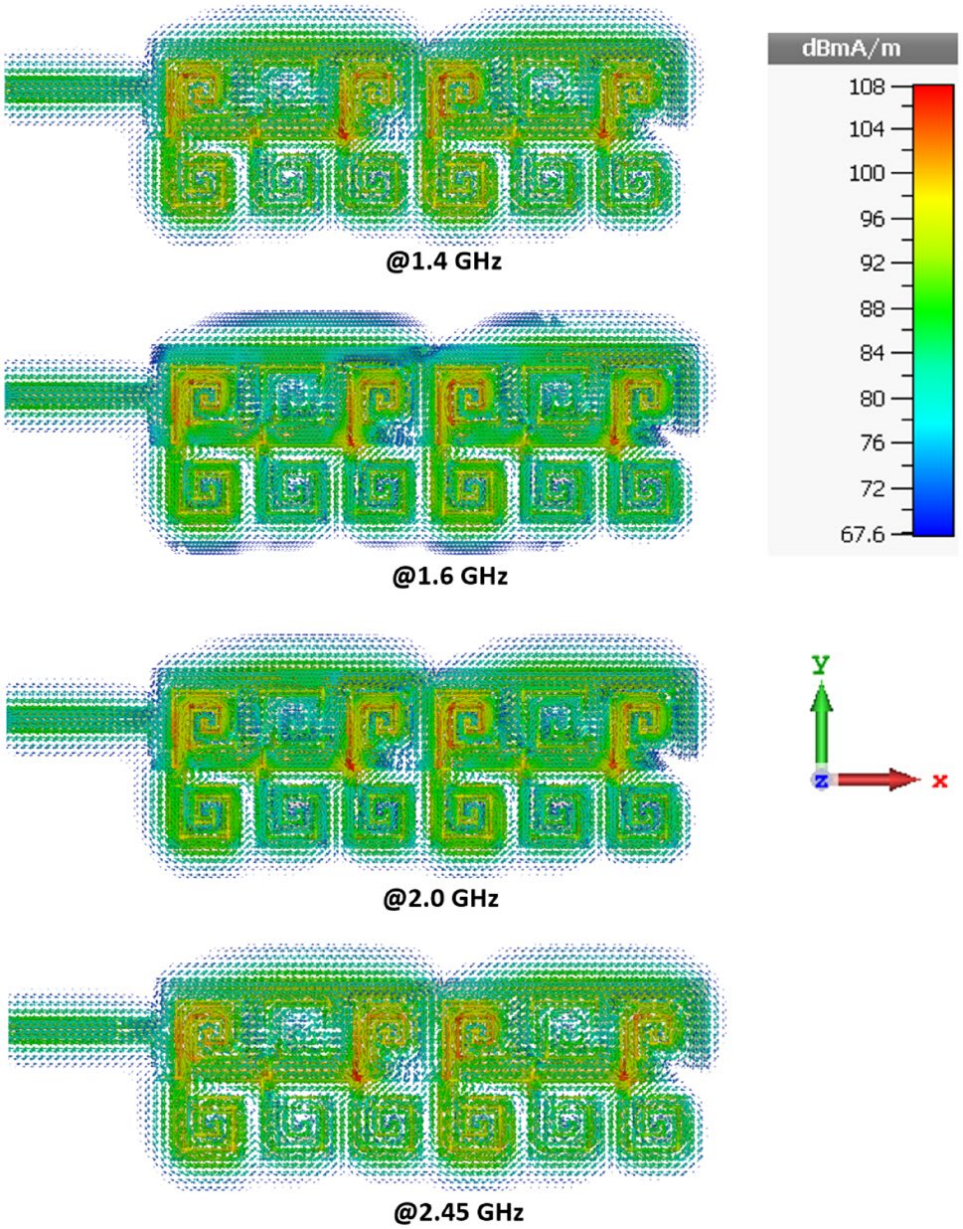
Current density distributions over the proposed antenna at spot frequencies of 1.4 GHz, 1.6 GHz, 2.0 GHz, and 2.45 GHz.

**Figure 13:**
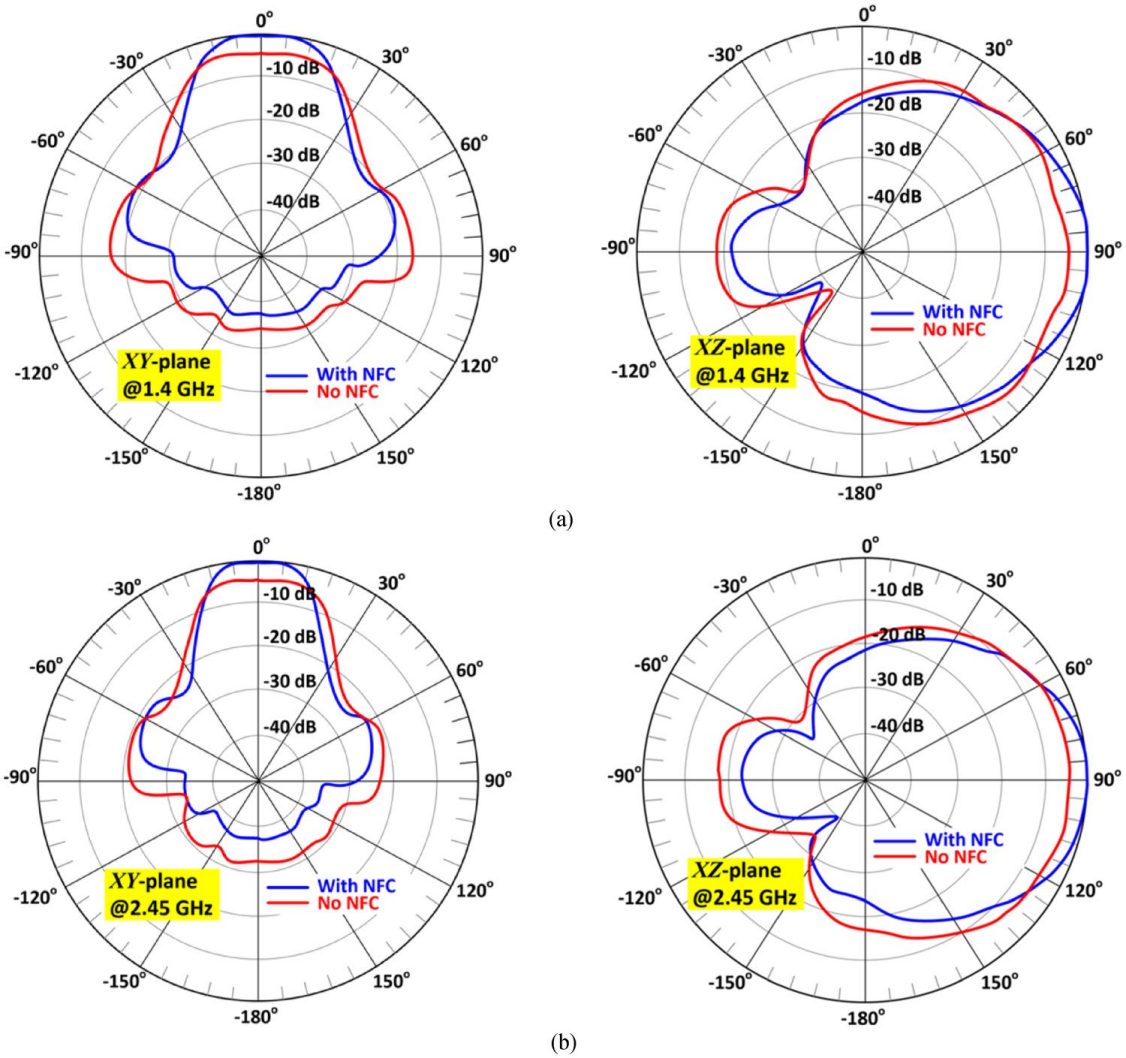
Measured radiation patterns (normalized) of the antenna without and with NF-IMC in the *xy*-plane (E-plane) and *xz*-plane (H-plane) at (**a**) 1.4 GHz and (**b**) 2.45 GHz.

Figure 12 shows how the current density is distributed over the surface of the antenna at various frequencies in its operating frequency band. It reveals the magnitude of the current is strongest over the middle two and outer unit-cells. The measured 2D radiation patterns of the antenna in the *xy*-plane (E-plane) and *xz-*plane (H-plane) with and without NF-IMC at 1.4 GHz and 2.45 GHz are shown in Fig. 13. The antenna’s radiation energy is mainly focused in the *x*-direction. The beamwidth is much narrower in the *xy*-plane (E-plane).

The performance of the proposed CRLH-TL antenna with NF-IMC is compared with other state-of-the-art antennas reported in literature. The results of this study are summarized in Table 3. The main advantages of the proposed work are its simplicity and stability over a very wide (> 1 GHz) operating frequency range.

**Table 3:** Performance Comparison Between the Proposed Work and Prior Arts.

References	FBW (%) [Freq. range]	Measured Gain (dBi)	Size (mm^3^)	NF-IMC topology
^18^	4.9 [400-420 MHz]	1.05	Not given	Relatively simple
^19^	25.6 [85–110 MHz]	3	73 × 50 × 1.6	Relatively simple
^20^	66.7 [80–160 MHz]	3	20 × 20 × 0.5	Requires 4 transistors
^21^	3.6 [1.35–1.45 GHz]	Not given	70 × 48 × 0.5	Requires transversal filter using distributed amplifiers & delay lines
^22^	20 [1.0–1.5 GHz]	5	12 × 12 × 1	Requires four transistors
^23^	26.1 [100–130 MHz]	Not given	Not given	Relatively simple
This work	54.5 [1.4–2.45 GHz]	6.55	56 × 20 × 0.8	Relatively simple

## Conclusions

It has been shown that by incorporating a non-Foster impedance matching circuit (NF-IMC) in-line with a miniature antenna, which is based on composite right/left-handed transmission-line technology, the operating bandwidth and radiation gain of the antenna can be substantially enhanced without affecting the antenna’s stability. This is because the negative reactance generated by the NF-IMC is used to counteract the input capacitance of the antenna. The results presented demonstrate the effectiveness of this technique where the antenna’s bandwidth is shown to increase from 380 MHz to greater than 1 GHz. Moreover, by incorporating the NF-IMC the average radiation gain and efficiency are shown to improve by 3.2 dBi and 31.5% over its operating bandwidth.
